# The chloroplast DNA locus *psbZ-trnfM* as a potential barcode marker in *Phoenix* L. (Arecaceae)

**DOI:** 10.3897/zookeys.365.5725

**Published:** 2013-12-30

**Authors:** Marco Ballardini, Antonio Mercuri, Claudio Littardi, Summar Abbas, Marie Couderc, Bertha Ludeña, Jean-Christophe Pintaud

**Affiliations:** 1 Centro Studi e Ricerche per le Palme - Sanremo (CSRP), Corso F. Cavallotti 113, I-18038 Sanremo (IM), Italy; 2 Consiglio per la Ricerca e la sperimentazione in Agricoltura - Unità di Ricerca per la Floricoltura e le Specie Ornamentali (CRA-FSO), Corso degli Inglesi 508, I-18038 Sanremo (IM), Italy; 3 Institute of Horticultural Sciences, University of Agriculture, 38040 Faisalabad, Pakistan; 4 UMR DIADE/DYNADIV, Institut de Recherche pour le Développement (IRD), 911 Av. Agropolis, F-34394 Montpellier Cedex 5, France

**Keywords:** Chloroplast *psbZ-trnfM* (CAU) region, DNA barcode, minisatellite, palms

## Abstract

The genus *Phoenix (Arecaceae)* comprises 14 species distributed from Cape Verde Islands to SE Asia. It includes the economically important species *Phoenix dactylifera*. The paucity of differential morphological and anatomical useful characters, and interspecific hybridization, make identification of *Phoenix* species difficult. In this context, the development of reliable DNA markers for species and hybrid identification would be of great utility. Previous studies identified a 12 bp polymorphic chloroplast minisatellite in the *trnG* (GCC)-*trnfM* (CAU) spacer, and showed its potential for species identification in *Phoenix*. In this work, in order to develop an efficient DNA barcode marker for *Phoenix*, a longer cpDNA region (700 bp) comprising the mentioned minisatellite, and located between the *psbZ* and *trnfM* (CAU) genes, was sequenced. One hundred and thirty-six individuals, representing all *Phoenix* species except *P. andamanensis*,were analysed. The minisatellite showed 2-7 repetitions of the 12 bp motif, with 1-3 out of seven haplotypes per species. *Phoenix reclinata* and *P. canariensis* had species-specific haplotypes. Additional polymorphisms were found in the flanking regions of the minisatellite, including substitutions, indels and homopolymers. All this information allowed us to identify unambiguously eight out of the 13 species, and overall 80% of the individuals sampled. *Phoenix rupicola* and *P. theophrasti* had the same haplotype, and so had *P. atlantica*, *P. dactylifera*, and *P. sylvestris* (the “date palm complex” *sensu* Pintaud et al. 2013). For these species, additional molecular markers will be required for their unambiguous identification. The *psbZ-trnfM* (CAU) region therefore could be considered as a good basis for the establishment of a DNA barcoding system in *Phoenix*, and is potentially useful for the identification of the female parent in *Phoenix* hybrids.

## Introduction

### Taxonomy and phylogeny of *Phoenix* L.

The genus *Phoenix* L. (*Arecaceae*) comprises 14 species (Govaerts and Dransfield 2005), distributed from the E Atlantic (Macaronesia), through Africa, the Mediterranean region, S Asia to islands in the Indian Ocean (Madagascar, Andaman) and the NW Pacific (Taïwan and N Philippines). *Phoenix* is morphologically and phylogenetically highly divergent from the other palm genera, and constitutes the monogeneric tribe *Phoeniceae* within the subfamily Coryphoideae (Asmussen et al. 2006, Dransfield et al. 2008). The position of *Phoenix* within the subfamily Coryphoideae has been confirmed by a generic-level phylogenetic analysis of the entire palm family (*Arecaceae*) that included plastid and nuclear DNA sequences, cpDNA RFLPs and morphological data (Baker et al. 2009).

The taxonomy, phylogeny and evolution of the genus itself have been assessed using morphological and molecular approaches. According to Barrow (1998), both morphological, and molecular data of the 5S intergenic spacer of the nuclear ribosomal 5S DNA unit supported the existence of two clades of closely related species. The first clade included *Phoenix dactylifera*, *Phoenix sylvestris*, *Phoenix theophrasti* and *Phoenix canariensis* –the so-called “date-palm complex”–, and *Phoenix atlantica* (Pintaud et al. 2010). The second group comprised the sister species *Phoenix paludosa* and *Phoenix roebelenii*. However, Barrow’s (1998) molecular analysis included only 11 out of the 13 species recognized at that time, since *Phoenix atlantica* was left as an insufficiently known taxon. Its status as a valid species was confirmed later by Henderson et al. (2006). Using one plastid and 16 nuclear microsatellite markers, Pintaud et al. (2010) demonstrated that all members of the “date-palm complex” are distinct species. Moreover, their data suggested that *Phoenix atlantica* and *Phoenix dactylifera* were sister species. Unfortunately, *Phoenix paludosa* and *Phoenix andamanensis* were not included in their analyses. Combining sequence data of the chloroplast *psbZ-trnfM* and *rpl16-rps* 3 loci, Pintaud et al. (2013) depicted five distinct phylogenetic lineages within *Phoenix* (*Phoenix loureiroi-acaulis-pusilla*, *Phoenix roebelenii-paludosa*, *Phoenix caespitosa*, *Phoenix reclinata*, and *Phoenix rupicola-theophrasti-canariensis-dactylifera-atlantica-sylvestris*), and restricted the “date palm complex” to *Phoenix dactylifera-atlantica-sylvestris*. This complex could be distinguished by the presence of a 3-repetitions haplotype of a 20 bp minisatellite motif at the *rpl* 16*-rps* 3 locus, that was absent in all other species. *Phoenix andamanensis* was the only taxon not included in their study.

The cultivated date palm *Phoenix dactylifera* L. is the most important fruit crop in the Middle East and North African countries. This species was probably domesticated around 4 000 B.C. in the Mesopotamia-Arabic Gulf area (Nesbitt 1993, Zohary and Hopf 2000, Tengberg 2012) and is nowadays distributed worldwide.

*Phoenix* species are largely interfertile and many interspecific hybrids have been recognized or suspected (Greuter 1967, Wrigley 1995). The spread of the domesticated *Phoenix dactylifera* resulted in situations of sympatry with wild species, promoting interspecific gene flow, in particular with the endemic *Phoenix canariensis* in the Canary Islands (González-Pérez et al. 2004), and possibly with *Phoenix theophrasti* in Turkey (Boydak and Barrow 1995), *Phoenix atlantica* in the Cape Verde Islands (Henderson et al. 2006), and *Phoenix sylvestris* in NW India (Newton et al. 2013). Moreover, spontaneous and directed hybridization between species is an important aspect of *Phoenix* ornamental cultivation (Tournay 2009).

Added to the common hybridization process between *Phoenix* species, the paucity of systematically useful morphological and anatomical characters within the genus (Barrow 1998), makes it difficult to establish a comprehensive taxonomy of the genus *Phoenix*. Because of this confusing situation, a reliable DNA marker set (barcode) to discriminate among *Phoenix* species and hybrids would be extremely useful.

### DNA barcoding

Hebert et al. (2003) introduced the concept of “DNA barcode” as a new approach to taxon recognition, assuming that a short standardised DNA sequence can distinguish individuals of a species because genetic differentiation between species exceeds that within species. Since then, DNA barcoding has become increasingly important as a tool in taxonomic studies and species delimitation, as well as in the discovery of new (cryptic) species (e.g. DeSalle et al. 2005, Hebert et al. 2004, Hebert and Gregory 2005, Savolainen et al. 2005, Hajibabaei et al. 2007). A consortium of scientists suggested the two-locus combination of *rbc*L + *mat*K plastid genes as the universal plant barcode (CBOL 2009), while other authors (Chen et al. 2010, Yao et al. 2010) proposed the ITS2 region as a more efficient barcode. The China Plant BOL Group (2011) highlighted the importance of both sampling multiple individuals and using markers with different modes of inheritance, and suggested to incorporate the ITS1/ITS2 region into the core barcode for seed plants.

However, despite all efforts, no locus (alone or in combination), has proven to be 100% efficient as universal DNA barcode in plants at the species level.

The first DNA barcoding analysis in palms (Jeanson et al. 2011) achieved a 92% success in species discrimination by applying a combination of three markers (the plastid *mat*K and *rbc*L, and the nuclear ITS2) to the tribe *Caryoteae* (subfamily Coryphoideae).

Investigating the taxonomic status of *Phoenix atlantica*, in comparison with its close relatives *Phoenix dactylifera*, *Phoenix canariensis* and *Phoenix sylvestris*, Henderson et al. (2006) identified a polymorphic cpDNA minisatellite locus, situated within the *trnG* (GCC)-*trnfM* (CAU) intergenic spacer. Its structure was based on the 12 bp motif CTAACTACTATA repeated in tandem 2-6 times. Four haplotypes were observed: one specific of *Phoenix canariensis*, one restricted to some individuals of *Phoenix sylvestris*, and two shared between *Phoenix dactylifera*, *Phoenix atlantica* and *Phoenix sylvestris*. Pintaud et al. (2010) studied this locus in 12 *Phoenix* species, identifying five haplotypes, whose pattern of variation was strongly associated with species. The maximum number of haplotypes per species was three (*Phoenix roebelenii*). Yet, most of the haplotypes were shared between species, viz. the 3-repetitions haplotype was the most common haplotype within the genus, and was shared by eight out of the 12 species. *Phoenix canariensis* was the only taxon characterised by the 5-repetitions haplotype. Hence, despite the promising information obtained, the minisatellite alone did not allow to distinguish all *Phoenix* species.

**Figure 1.**
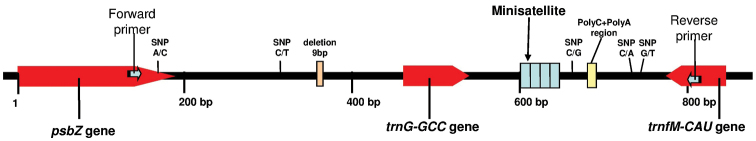
The sequenced cpDNA *psbZ-trnfM* region.The location of PCR primers used and polymorphisms found in this study are shown. DNA fragment length refers to the *Phoenix dactylifera cv. Khalas* cpDNA sequence (Yang et al. 2010), characterised by a 4-repetitions minisatellite haplotype (NCBI Reference Sequence: NC_013991.2).

Given the potential of the *trnG* (GCC)*-trnfM* (CAU) spacer for barcoding in *Phoenix*, we examined a wider cpDNA region, viz. a ~700 bp sequence *psbZ-trnfM* (CAU) (Figure 1), in search of an efficient DNA barcode locus for species delimitation and identification of female parents in hybrids in the genus *Phoenix*.

## Methods

### Taxon sampling

One hundred and thirty-six individuals, belonging to 13 *Phoenix* species, with emphasis on *Phoenix dactylifera*, were analysed in this work (Appendix). *Phoenix andamanensis* was not included in the analysis due to a lack of material.

### DNA sequencing

For each sample, genomic DNA was extracted from 40 mg of freeze-dried leaf tissue which was first grinded using a bead-mill homogenizer Tissuelyser (Qiagen, France). Extraction was performed using the DNeasy Plant Mini Kit protocol along with the QIAcube robotic workstation for DNA automated purification (Qiagen, France). Extracted DNA was quantified by means of a Nanodrop ND1000 spectrophotometer (Thermo Fisher Scientific Inc., USA) and visualized on 1% agarose gels stained with ethidium bromide.

The PCR amplification was carried out using the monocotyledoneous universal primers *psbZ*-IGS-F: GGTACMTCATTATGGATTGG, and *trnfM*-IGS-R: GCGGAGTAGAGCAGTTTGGT (Scarcelli et al. 2011). The amplified cpDNA fragment was approximately 700 bp long. PCR reactions were prepared in 25 µl of total volume, containing the following reagent concentrations: 5 ng/µl DNA template, 0.2 μM each of forward and reverse primers, 2X Failsafe PCR PreMix E (Epicentre Biotechnologies, Madison USA), 2.5 U/µl Failsafe Enzyme Mix (Epicentre Biotechnologies, USA), and DNase-free sterile water. PCR parameters were the following: an initial denaturation step at 94 °C for 3 min, then 35 cycles at 94 °C for 30 s, 60 °C for 30 s, and 72 °C for 1 min, and a final elongation step at 72 °C for 10 min. PCR products were controlled on 1% agarose gels stained with ethidium bromide and then purified using Ampure Agencourt kit (Agencourt Bioscience Corporation, USA). Their quantification was done by means of a Nanodrop ND1000 spectrophotometer (Thermo Fisher Scientific Inc., USA). Cycle sequencing was carried out using the Big Dye Terminator v3.1 kit (Applied Biosystems, USA). Cycle sequencing products were purified using the CleanSeq Agencourt Kit (Agencourt Bioscience Corporation, USA) and were then analysed on an ABI 3130 automated DNA Sequencer (Applied BioSystems, USA).

### Sequence alignment and identification success

The chromatograms obtained with the forward and reverse primers were combined and edited with SeqMan II 5.00 software (DNASTAR Inc., USA), to generate consensus sequences, which were aligned in BioEdit (Hall 1999), using the Clustal W algorithm. The obtained alignment was further improved manually with MESQUITE v2.75 (Maddison and Maddison 2007). The observed polymorphisms were positioned in reference to the complete chloroplast genome sequence of *Phoenix dactylifera cv. Khalas*, available in GenBank (accession NC_013991.2).

To assess the potential of the *psbZ*-*trnfM* region as a barcode for accurate species identification, we evaluated the proportion of correct identifications using TaxonDNA (Meier et al. 2006). The Best Match and Best Close Match tests were run for species with > 1 individual and with nearly complete sequences, which resulted in a reduced dataset of 11 species (excluding *Phoenix acaulis* and *Phoenix atlantica*) and 121 individuals. Because of this constraint, the two species represented by only one individual were analysed by direct comparison of their sequences. Moreover, direct sequence comparison included not only nucleotide substitutions as in the TaxonDNA analysis, but also indels, minisatellites and homopolymers.

## Results

The amplification of the plastid target region *psbZ-trnfM* (CAU) was successful for all samples, and the sequencing with both primers was achieved for 123 individuals, while a single read (forward or reverse) was retrieved for the other 13 individuals, whose sequences were approximately 20% shorter.

The analysis of the intra- and interspecific variation within the sequenced region by direct observation of the sequence alignment showed four mutation types that contributed to the separation of *Phoenix* species: single nucleotide polymorphisms (SNPs), indels, length variation at the 12 bp minisatellite locus, and in homopolymers, allowing in total to identify unambiguously eight out of the 13 species (Table 1).

**Table 1.** Distribution of observed polymorphisms in the region *psbZ-trnfM* (CAU)

Substitutions^a^					9 bp deletion^a^	Minisatellite^c^	Homo-polymer^a^	Species^b^
36607	36754	37099	37183	37190	36795-36803	37050-37098	37128-37139	
Haplotypes recorded in a single species^d^ (80.1% total sampling)								
C	T	G	A	T	absent	**5M1+1M2**^(4)^	7 C + 5 A	*Phoenix canariensis* (7)
C	T	**C**	A	T	absent	**2M1+5bp+1M2**^(6)^	**6 C + 5 A**	*Phoenix reclinata* (4)
C	T	G	A	T	absent	**1M1+2M2**^(7)^	7 C + 5 A	*Phoenix reclinata* (6)
C	T	G	**C**	T	absent	6M1+1M2^(5)^	7 C + 5 A	*Phoenix caespitosa* (2)
C	C	G	A	**G**	absent	2M1+1M2^(1)^	7 C + 5 A	*Phoenix loureiroi* (1)
A	C	G	A	T	absent	3M1+1M2^(2)^	6 C + 6 A	*Phoenix loureiroi* (1)
A	C	G	A	T	absent	2M1+1M2^(1)^	7 C + 5 A	*Phoenix acaulis* (1)
C	C	G	A	T	absent	2M1+1M2^(1)^	6 C + 6 A	*Phoenix pusilla* (2)
C	T	G	A	T	present	2M1+1M2^(1)^	6 C + 6 A	*Phoenix paludosa* (2)
C	T	G	A	T	present	4M1+1M2^(3)^	**5 C + 7 A**	*Phoenix roebelenii* (3)
C	T	G	A	T	present	3M1+1M2^(2)^	**5 C + 7 A**	*Phoenix roebelenii* (1)
C	T	G	A	T	absent	4M1+1M2^(3)^	7 C + 5 A	*Phoenix dactylifera* (78)
C	T	G	A	T	absent	2M1+1M2^(1)^	**8 C + 5 A**	*Phoenix sylvestris* (1)
Haplotypes shared by two species (5.1%)								
C	T	G	A	T	absent	6M1+1M2^(5)^	7 C + 5 A	*Phoenix rupicola* (3)
								*Phoenix theophrasti* (4)
Haplotypes shared by three species (14.8%)								
C	T	G	A	T	absent	3M1+1M2^(2)^	7 C + 5 A	*Phoenix atlantica* (1)
								*Phoenix dactylifera* (16)
								*Phoenix sylvestris* (3)

^a^ Position in the complete chloroplast genome of *Phoenix dactylifera* ‘Khalas’ accession NC_013991.2.

^b^ Number of individuals analysed for each species in parentheses (total sampling of 136 specimens).

^c^ Number of repetitions of the 12 bp minisatellite units, including number of units of motif 1 (M1) and motif 2 (M2) as represented in Figure 2.

^d^ Species-specific mutations in bold.

^(1–7)^ Minisatellites haplotypes as reported in Figure 2 (1 to 7).

**Figure 2.**
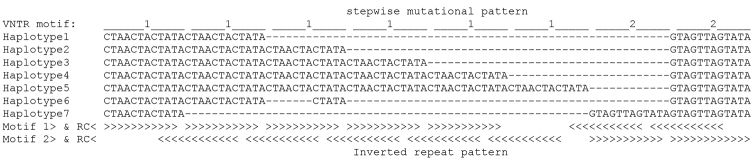
Structure and variation of the minisatellite in the *trnG-trnfM* intergenic spacer. The repeats of the two mutational motifs (1 and 2) are indicated above the sequence alignment of the 7 haplotypes recorded. The pattern of inverted repeats generated by the two motifs and their reverse complements (RC) is shown below the alignment. See Table 1 for haplotype distribution among species.

The minisatellite located in the *trnG* (GCC)*-trnfM* (CAU) intergenic spacer showed seven haplotypes. Most haplotypes corresponded to a Variable Number Tandem Repeat (VNTR) stepwise mutational pattern of 12 bp units. These units corresponded to two motifs: CTAACTACTATA (motif 1) and GTAGTTAGTATA (motif 2), which form between themselves a pattern of 12 bp inverted repeats shifted with respect to the boundaries of the mutational units (Figure 2). One haplotype, found in four out of ten *Phoenix reclinata* individuals, departed from this pattern, with two complete units of motif 1 plus an incomplete third unit with a 7 bp-deletion (CTAACTA) (haplotype 6; Figure 2). These four specimens were further characterized by a SNP (C instead of G) at position 37099. The other six *Phoenix reclinata* samples were also unique in having two repeats of motif 2 instead of one as in the six other haplotypes (haplotype 7; Figure 2). *Phoenix canariensis* was characterised by a private haplotype with five repeats of motif 1 (haplotype 4; Figure 2). The maximum number of haplotypes per species was three; in that case, one or two of them were shared by different species (Table 1).

Additional deletions and SNPs were detected in some of the analysed species, both upstream and downstream of the minisatellite (Table 1). *Phoenix roebelenii* and *Phoenix paludosa* shared a 9 bp-deletion (GTACTTTAC, upstream to the minisatellite, in position 36795-36803), *Phoenix acaulis*, *Phoenix loureiroi*, and *Phoenix pusilla* shared a SNP at position 36754 (C instead of T), while *Phoenix caespitosa* had a SNP at position 37183 (C instead of A). One of the three samples of *Phoenix loureiroi* showed a SNP in position 37190 (G instead of T), and another sample shared a SNP with the single specimen of *Phoenix acaulis* (A instead of C in position 36607). Some more differences among species and/or individuals were found in an homopolymer region (C_n_ + A_n_), located at position 37128-37139, downstream to the minisatellite (Table 1).

*Phoenix theophrasti* and *Phoenix rupicola* shared the 6 repetitions of motif 1 minisatellite haplotype (haplotype 5; Figure 2) and could not be distinguished from each other (*Phoenix caespitosa* had also the 6 repetitions haplotype but was further differentiated by a species-specific SNP). Within the “date palm-complex” (Pintaud et al. 2013), a 3 repetitions of motif 1 haplotype was shared by some specimens of *Phoenix atlantica*, *Phoenix dactylifera*, and *Phoenix sylvestris* (haplotype 2; Figure 2), while a majority of individuals of *Phoenix dactylifera* were uniquely identified by a 4 repetitions of motif 1 haplotype (haplotype 3; Figure 2).

The TaxonDNA pairwise comparison analysis of the 121 samples retained resulted in 115 sequences with a closest match at 0%. There were 18 allospecific matches at 0% (15.65%). At the individual level, the Best Match test, and the Best Close Match test with a threshold of 3%, resulted in 82.64% correct identifications, 14.87% ambiguous identifications and 2.47% incorrect identifications. At the species level, however, only *Phoenix caespitosa* could be unambiguously identified, since it was the only species in the sampling with an autapomorphic SNP.

The haplotype sequences used, and the new ones obtained during this study, are deposited in GenBank under accessions: JF745571, EU043486, EU043484, EU043485, JX970915–JX970936.

## Discussion

In this study, we tested the usefulness of the *psbZ-trnfM* (CAU) region as a barcode locus in *Phoenix*. The successful amplification and sequencing of this marker within all of the analysed species confirms its value in terms of universality. Moreover, its high performance should allow the acquisition of barcode information even with partially degraded DNA samples.

TaxonDNA unambiguously identified a single species, *Phoenix caespitosa*, due to the scarcity of SNPs, most of them shared by two or more species, or on the contrary restricted to a subset of individuals within species. Therefore, it is important to take into account the other polymorphisms (indels, minisatellites and homopolymers) which usually represent half or more of the mutations in non-coding chloroplast DNA (Scarcelli et al. 2011). However, at the individual level, the Best Match and Best Close Match tests resulted in more than 80% correct identifications, which is indicative of the barcoding potential of the marker studied.

The 9 bp-deletion, shared by *Phoenix roebelenii* and *Phoenix paludosa*, supports Barrow’s conclusions (1998), as well as Pintaud et al.’s (2013), regarding the close relationship between these two taxa.

Regarding the 12 bp minisatellite, our results revealed much more complexity than previously reported (Pintaud et al. 2010). This could be explained by the increased sampling of the present study, and also by differences in methodology, i.e. sequencing versus genotyping. In particular, the genotyping data of Pintaud et al. (2010) did not detect the 7 bp-deletion found within the minisatellite of some *Phoenix reclinata* samples, and were also misled by the size homoplasy between haplotype 1 and 7 (Figure 2). We therefore recommend that sequence data should be obtained before performing any study based on genotyping, in order to have a solid basis to interpret genotyping data.

In total, considering all mutation types, our results allowed us to efficiently identify eight out of 13 species. This indicates that the locus *psbZ-trnfM* (CAU) has some potential to yield DNA barcodes that can be used for species identification within the genus *Phoenix*. This locus could also be useful to identify the female parent in many interspecific crosses, such as *Phoenix dactylifera* × *Phoenix canariensis*. Hybrids involving *Phoenix canariensis* as female parents are particularly easy to track because this species is monomorphic with a private haplotype at the locus studied. Hybrids between these two species are a concern for the genetic integrity of native populations of *Phoenix canariensis* in the Canary Islands (González-Pérez et al. 2004). Such hybrids are also very common in ornamental plantings, for which they represent a valuable horticultural resource.

Nevertheless, in order to increase resolution, other DNA regions should be examined, in search of characters allowing the identification of all taxa. Given their proven utility in palms, the *psbA-trnH* locus (Al-Qurainy et al. 2011) and/or the ribosomal ITS2 (Jeanson et al. 2011) could be investigated in combination with *psbZ-trnfM* for this purpose. Special attention should be paid to the species group sharing haplotype 2 (Figure 2): *Phoenix atlantica*, *Phoenix dactylifera* and *Phoenix sylvestris*. This group is composed of very closely related species, so difficulty in DNA barcoding for these species is expected. On the other hand, in some cases, the morphological divergence is not associated to sequence divergence in the *psbZ-trnfM* region. For example, *Phoenix rupicola* and *Phoenix theophrasti* share the same haplotype despite considerable morphological differentiation and geographical isolation, the former being restricted to the E Himalayan, while the latter is an Aegean endemic. These two species possibly share plesiomorphic SNP states and may show convergence in the minisatellite haplotype. In contrast, *Phoenix dactylifera* and *Phoenix theophrasti* are phenotypically very similar, but can easily be distinguished at the *psbZ-trnfM* (CAU) region. The relation between morphological divergence and molecular divergence at the *psbZ-trnfM* (CAU) region among the *Phoenix* species needs to be addressed with a larger sampling within species as recommended by the China Plant BOL Group (2011).

## Appendix

**List of samples.** DNA bank reference: IRD = Institut de Recherche pour le Développement, 911 Av. Agropolis, F-34394 Montpellier Cedex 5, France. Tissue bank reference: CRA-FSO = Consiglio per la Ricerca e la sperimentazione in Agricoltura - Unità di Ricerca per la Floricoltura e le Specie Ornamentali, Corso degli Inglesi 508, I-18038 Sanremo (IM), Italy.

***Phoenix acaulis* Roxb**: MWC5559, Kew, UK (IRD). ***Phoenix atlantica* A. Chev.**: SH25, Cape Verde (IRD). ***Phoenix caespitosa* Chiov.**: MWC1195, MWC1802, Kew, UK (IRD). ***Phoenix canariensis* Chabaud**: 93.100, 93.101, 93.103, 93.107, Sanremo, Italy (IRD, CRA-FSO); MWC1396, Kew, UK (IRD); JCP169, JCP210, Canary Isl., Spain (IRD). ***Phoenix dactylifera* L.**: 93.003, 93.004, 93.005, 93.025, 93.027, 93.030, 93.037, 93.043, 93.045, 93.047, 93.048, 93.049, 93.052, 93.054, 93.055, 93.056, 93.059, 93.060, 93.061, 93.065, 93.066, 93.067, 93.070, 93.071, 93.072, 93.073, 93.076, 93.077, 93.080, 93.085, 90.002, 90.003, 90.004, 90.005, 90.006, 90.007, 90.008, 90.009, 90.010, 90.011, 90.012, 90.013, 90.014, 90.015, 90.025, 90.026, 90.027, 90.028, 90.029, 91.005, Sanremo, Italy (IRD, CRA-FSO); 00.01, 00.02, 00.03, 00.04, 00.05, 00.06, 00.07, 00.08, 00.09, 00.10, 00.11, 00.13, 00.14, 00.83, 00.85, 00.88, 46.02, 46.04, 46.05, 46.06, 46.08, 46.09, 46.14, 46.15, 46.16, 46.17, 46.18, 46.19, 46.20, 46.21, 46.23, JCP413, JCP414, JCP415, JCP416, JCP417, Bordighera, Italy (IRD, CRA-FSO); DAT077-365, DAT079-366, Oman (IRD); JCP260, Murcia, Spain; JCP426, Elche, Spain; SZ1, SZ2, SZ5, SZ10, Tunisia (IRD). ***Phoenix loureiroi* Kunth var. *loureiroi***: JCP409, Montgomery Botanical Garden, Miami, USA (IRD); MWC1187, Kew, UK (IRD). ***Phoenix paludosa* Roxb.**: MWC1190, MWC1877, Kew, UK (IRD). ***Phoenix pusilla* Gaertn.**: JCP213_5, Sri Lanka (IRD); MWC1806, Kew, UK (IRD). ***Phoenix reclinata* Jacq.**: ECH3-A, ECH4-A, ECH5-B, 91.001, 91.007, 91.008, 91.009, 91.033, 92.003, Sanremo, Italy (IRD, CRA-FSO); MWC1397, Kew, UK (IRD). ***Phoenix roebelenii* O’Brien**: ECH1-A, ECH2-A, Sanremo, Italy (IRD, CRA-FSO); MWC1400, MWC1805, Kew, UK (IRD). ***Phoenix rupicola* T. Anderson**: ECH6-A, ECH8-A, Sanremo, Italy (IRD, CRA-FSO); MWC1399, Kew, UK (IRD). ***Phoenix sylvestris* (L.) Roxb.**: DAT057-345, Elche, Spain (IRD); JCP214, The Palm Center, UK, (IRD); JCP405-388, Thuret, France (IRD); MWC1876, Kew, UK (IRD). ***Phoenix theophrasti* Greuter**: ECH7-A, ECH9-A, Sanremo, Italy (IRD, CRA-FSO); JCP215, The Palm Center, UK (IRD); MWC1163, Kew, UK.
